# Dynamic fluctuations of intrinsic brain activity are associated with consistent topological patterns in puberty and are biomarkers of neural maturation

**DOI:** 10.1162/netn_a_00452

**Published:** 2025-09-19

**Authors:** Jethro Lim, Kaitlynn Cooper, Catherine Stamoulis

**Affiliations:** Department of Pediatrics, Division of Adolescent and Young Adult Medicine, Boston Children’s Hospital, Boston, MA, USA; Carnegie Mellon University, Pittsburgh, PA, USA; Department of Pediatrics, Harvard Medical School, Boston, MA, USA

**Keywords:** Developing brain, Connectome, Intrinsic dynamics, Resting-state topology, Fluctuation amplitude

## Abstract

Intrinsic brain dynamics play a fundamental role in cognitive function, but their development is incompletely understood. We investigated pubertal changes in temporal fluctuations of intrinsic network topologies (focusing on the strongest connections and coordination patterns) and signals, in an early longitudinal sample from the Adolescent Brain Cognitive Development (ABCD) study, with resting-state fMRI (*n* = 4,099 at baseline; *n* = 3,376 at follow-up [median age = 10.0 (1.1) and 12.0 (1.1) years; *n* = 2,116 with both assessments]). Reproducible, inverse associations between low-frequency signal and topological fluctuations were estimated (*p* < 0.05, *β* = −0.20 to −0.02, 95% confidence interval (CI) = [−0.23, −0.001]). Signal (but not topological) fluctuations increased in somatomotor and prefrontal areas with pubertal stage (*p* < 0.03, *β* = 0.06–0.07, 95% CI = [0.03, 0.11]), but decreased in orbitofrontal, insular, and cingulate cortices, as well as cerebellum, hippocampus, amygdala, and thalamus (*p* < 0.05, *β* = −0.09 to −0.03, 95% CI = [−0.15, −0.001]). Higher temporal signal and topological variability in spatially distributed regions were estimated in girls. In racial/ethnic minorities, several associations between signal and topological fluctuations were in the opposite direction of those in the entire sample, suggesting potential racial differences. Our findings indicate that during puberty, intrinsic signal dynamics change significantly in developed and developing brain regions, but their strongest coordination patterns may already be sufficiently developed and remain temporally consistent.

## Author Summary

We have investigated pubertal changes in intrinsic signal and network dynamics, estimated from resting-state fMRI in a sample of youth from the ABCD study. We have identified reproducible, inverse associations between low-frequency signal and topological fluctuations, as well as pubertal changes in intrinsic signal dynamics but not topological patterns of strongly connected networks. We have also identified sex differences in these dynamics and negative associations with body mass index (BMI). Several associations between signal and topological fluctuations were in the opposite direction in racial/ethnic minorities compared with those in the entire sample. Our findings indicate that intrinsic signal dynamics change significantly in developed and developing brain regions during puberty, but their strongest synchronization patterns may already be sufficiently developed prior to puberty and are dynamically reproducible.

## INTRODUCTION

Even when not actively processing external inputs or responding to cognitive task demands, the brain is not at rest. Its activity varies dynamically at multiple temporal and spatial scales (as a function of its physiological and cognitive state). The origin and cognitive correlates of dynamically varying, intrinsic activity and spontaneous coordination of brain regions have been studied extensively in the human brain (Allen et al., 2014; Calhoun et al., 2014; Fox et al., 2006; Fox & Raichle, 2007; Fransson, 2005; Grady et al., 2023; Greicius et al., 2003; Krishan et al., 2018; Vidaurre et al., 2017; Vincent et al., 2007; Wise et al., 2004; Zhang & Northoff, 2022). Prior work has correlated intrinsic activity with cognitive processing at that state, including mental imagery, introspection (Gonzalez-Castillo et al., 2021), and mind wandering (Chou et al., 2017; Christoff et al., 2009; Mason et al., 2007). Studies on spontaneous network coordination have provided insights into temporal anticorrelations between brain regions with antagonistic functions (Fox & Raichle, 2007) and/or correlated temporal patterns that may reflect specialized cognitive function (Smith et al., 2013). Some have also linked aberrant spontaneous coordination patterns to neuropsychiatric disorders, such as schizophrenia (Damaraju et al., 2014; Fu et al., 2018; Northoff & Duncan, 2016), as well as cognitive decline and impairment (Lin et al., 2018; Meghdadi et al., 2021).

A number of studies have specifically investigated dynamic functional connectivity (dFC) using neuroimaging modalities with different spatiotemporal resolutions (e.g., fMRI and EEG), as well as different computational approaches (Cohen, 2018; Hutchison et al., 2013; Lurie et al., 2020; Preti et al., 2017). These include sliding window-based methods that calculate dFC in temporally overlapping windows using pairwise correlation or coherence measures and/or independent component analysis (ICA) and related approaches (Allen et al., 2014; Preti et al., 2017). Variants have also used an adaptive window, informed by local brain dynamics. Some studies have used instantaneous phase synchronization for high-resolution temporal estimation of coordination that is not sensitive to the window size (Fransson & Strindberg, 2023; Glerean et al., 2012), recurring patterns of voxel or region coordination (Liu et al., 2018), or sequences of recurring states (Vidaurre et al., 2017). ICA has also been used to identify multistate functional domains and track their spatial variation over time (Iraji, Fu, et al., 2019).

Relatively fewer studies have examined resting-state dynamics and dFC in the developing brain. They have identified age-related changes in these dynamics (Faghiri et al., 2018; Lei et al., 2022; López-Vicente et al., 2021) and multiple intrinsic connectivity states with distinct topological and temporal characteristics (e.g., duration and variability) that may be associated with introspective processes (Marusak et al., 2017), behavior and cognitive performance (Di et al., 2023; Ye et al., 2024), trait mindfulness (Marusak et al., 2018; Treves et al., 2024), and mental health (Fu et al., 2018, 2021, 2025). These, likely metastable, states facilitate the brain’s recruitment of task-related networks and transitions between patterns of coordination in response to cognitive demands (Deco et al., 2017) or from an introspective to an extrospective mode (Fransson, 2005). In addition, higher temporal variability of resting-state brain signals has been associated with better cognitive performance and social emotional health (Garrett et al., 2011; Grady et al., 2023). Higher dFC similarity (lower temporal and/or cross-scan variability) has been associated with better cognitive performance in both adults (Cabral et al., 2017) and children (Fu et al., 2023). Furthermore, mental health disorders, such as depression and bipolar disorder, have been linked to alterations in dFC variability. Youth with attention-deficit/hyperactivity disorder and autism spectrum disorders also have differences in dFC (Ahmadi et al., 2021; de Lacy et al., 2017; de Lacy & Calhoun, 2018; Luo et al., 2023).

Beyond dFC, other measures of spontaneous brain activity fluctuations have been estimated across the lifespan. A recent study has shown that the amplitude of low-frequency BOLD fluctuations and its spatial gradient may be a hallmark characteristic of cortical maturation and developmental changes in neural plasticity (Sydnor et al., 2023). Also, abnormally high variability in fluctuation amplitude has been correlated with generalized anxiety disorder symptoms (Cui et al., 2020; Shen et al., 2020). A few studies have also examined the temporal variability of resting-state topological network properties. Higher variability of functional network modularity (i.e., community structure) across the brain has been associated with periods of statistically unexpected fluctuations (Betzel et al., 2016). Fluctuations in the modularity of the dorsal attention network have been correlated with higher composite intelligence (Hilger et al., 2020).

Despite invaluable insights provided by prior studies, developmental (including pubertal) changes in intrinsic brain dynamics—as the brain’s anatomy, morphology, and circuit organization change extensively during almost 2 decades of life—remain incompletely understood. In particular, brain dynamics during complex periods of development such as adolescence (and especially puberty), which are associated with profound physical, biochemical, endocrine, and cognitive changes, have not been studied. Robust characterization of resting-state brain dynamics during this period is challenging, largely because of the high heterogeneity of adolescent brain development and unique environmental and experiential factors that play a critical role in shaping the brain’s unique wiring, and consequently its intrinsic networks and their organization. In addition, measures of temporal variability are inherently sensitive to the underlying brain dynamics, which also contribute to their heterogeneity. Thus, large cohorts are necessary to robustly estimate time-varying, resting-state network topologies and regional intrinsic activity in youth. Furthermore, longitudinal studies can provide critical insights into how this variability changes as a function of development, including pubertal maturation. Finally, studies that go beyond dFC and examine dynamic topological measures or resting-state networks are also needed, as they may provide important insights into the spontaneous organization (instead of just strength of connections provided by dFC) of resting-state networks, including their community structure, topological stability, efficiency, and resilience.

Large-scale studies, such as the longitudinal Adolescent Brain Cognitive Development (ABCD) study (Casey et al., 2018), provide unique opportunities to address this gap in knowledge and robustly characterize spontaneous neural dynamics and topological coordination in the developing human brain. A recent study investigated cognitive and mental health correlates of dFC states in the ABCD cohort (Fu et al., 2025), but did not specifically focus on changes in intrinsic signal and network dynamics during puberty. Other studies in independent cohorts have reported age-related changes over longer periods of development (Faghiri et al., 2018), but have not focused on adolescence and/or puberty. Also, most of these studies have not focused on both dynamically varying networks and intrinsic signal fluctuations together, and their relationships.

To address this gap in knowledge, the present study leveraged early longitudinal resting-state fMRI data from the ABCD to investigate pubertal changes in intrinsic dynamics. Specifically, in overlapping samples of over 4,000 youth at the ABCD baseline (ages ~9–10 years) and ~3,000 at the 2-year follow-up (ages ~11–12 years), it aimed to robustly characterize temporal fluctuations of spontaneous network topologies (and their properties) and BOLD signal fluctuation amplitude as a function of pubertal stage. The study hypothesized that the dynamics of resting-state networks and amplitude of spontaneous regional activity change significantly during puberty, largely as a result of heightened neural maturation (and associated changes in anatomical connections and morphometric characteristics) and underlying biochemical changes. It further hypothesized that the dynamic similarity (decreased variability) of resting-state network topologies increases as a function of development and likely reflects the maturation of their underlying anatomical constraints and increased stereotypy of spontaneous synchronization patterns.

## RESULTS

At baseline, almost half of youth were in prepuberty (*n* = 1,962 [47.9%]), 924 (22.5%) in early puberty, and 1,023 (25.0%) in mid-puberty. At follow-up, less than 20% were in prepuberty (*n* = 602 [17.8%]), 724 (21.4%) in early puberty, 1,116 (33.0%) in mid-puberty, and ~22.0% in later stages. The race and ethnicity distribution of the samples reflected the demographic characteristics of the ABCD cohort, which is predominantly White and non-Hispanic. In both the baseline and follow-up samples, about half of youth were White and non-Hispanic (2,020 [49.3%] and 1,728 [51.2%], respectively); ~15% were Black non-Hispanic, and similarly for other non-Hispanic racial groups; and less than 25% were Hispanic (932 [22.7%] and 756 [22.4%], respectively). The median participant BMI was 17.4 kg/m^2^ (interquartile range [IQR] = 4.6 kg/m^2^) at baseline and 19.3 kg/m^2^ (IQR = 5.5 kg/m^2^) at follow-up. Detailed demographic and other participant characteristics are provided in Table 1.

**Table 1.** Participant characteristics and demographic information in the baseline and 2-year follow-up samples

		**Baseline (*N* = 4,099)**	**Two-year follow-up (*N* = 3,376)**
**Sex**	**Male**	1,912 (46.65%)	1,650 (48.87%)
	**Female**	2,184 (53.28%)	1,724 (51.07%)
**Race/ethnicity**	**White Non-Hispanic**	2,020 (49.28%)	1,728 (51.19%)
	**Black Non-Hispanic**	609 (14.86%)	491 (14.54%)
	**Asian Non-Hispanic**	100 (2.44%)	70 (2.07%)
	**Other (including mixed race) non-Hispanic**	416 (10.15%)	321 (9.51%)
	**Hispanic**	932 (22.74%)	756 (22.39%)
	**Missing**	22 (0.54%)	10 (0.30%)
**Family income**	**<5,000**	124 (3.03%)	75 (2.22%)
	**5,000–24,999**	367 (8.95%)	255 (7.55%)
	**25,000–49,999**	541 (13.20%)	398 (11.79%)
	**50,000–99,999**	1,000 (24.40%)	844 (25.00%)
	**100,000–199,999**	1,211 (29.54%)	1,049 (31.07%)
	**>= 200,000**	512 (12.49%)	490 (14.51%)
	**Missing**	344 (8.39%)	265 (7.84%)
**Parental education**	**Advanced degree (MS, JD, MD, other professional)**	1,125 (27.45%)	958 (28.38%)
	**Bachelor’s degree**	1,183 (28.86%)	960 (28.44%)
	**Associate degree**	457 (11.15%)	420 (12.44%)
	**Some college**	669 (16.32%)	528 (15.64%)
	**High school/GED**	378 (9.22%)	294 (8.71%)
	**Did not graduate high school**	284 (6.93%)	210 (6.22%)
	**Missing**	3 (0.07%)	6 (0.18%)
**BMI (median [IQR])**	**Raw score**	17.54 (4.59)	19.34 (5.54)
	***z*-score**	−0.31 (1.11)	−0.053 (0.153)
	**Missing**	11 (0.27%)	22 (0.65%)
**Pubertal stage**	**Prepuberty**	1,962 (47.87%)	602 (17.83%)
	**Early puberty**	924 (22.54%)	724 (21.45%)
	**Mid-puberty**	1023 (24.96%)	1,116 (33.06%)
	**Late/postpuberty**	61 (1.49%)	761 (22.54%)
	**Missing**	129 (3.15%)	173 (5.12%)

### Topological and Amplitude Fluctuations in Early Longitudinal Sample

#### Spatial scale of individual regions.

**Figure 1.**
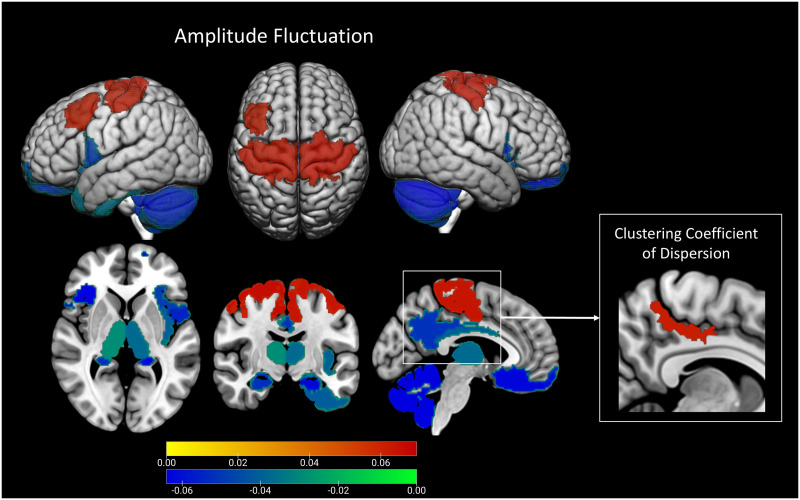
Spatial distribution of regions in which pubertal stage is statistically correlated with fluctuations in local clustering (left) and fluctuation amplitude (right). Colors correspond to standardized regression coefficients (negative: blue to green, positive: yellow to red).

Temporal fluctuations of local clustering (i.e., a region’s neighborhood connectedness) in parts of the right medial parietal cortex (a key region of the salience network involved in multiple processes including spatial navigation and awareness, self-processing, and social function) increased with pubertal stage (*p* < 0.02, *β* = 0.062, 95% CI = [0.022, 0.102]). In addition, fluctuation amplitude in the bilateral somatomotor cortices and left lateral prefrontal cortex also increased with pubertal stage (*p* < 0.03, *β* = 0.06–0.07, 95% CI = [0.03, 0.11]), but decreased in the left temporal pole, bilateral orbitofrontal cortex, insula, parahippocampal cortex, left posterior cingulate cortex, and bilateral subcortical areas including the cerebellum, hippocampus, amygdala, and thalamus (*p* < 0.05, *β* = −0.09 to −0.03, 95% CI = [−0.15, −0.001]), indicating increased temporal consistency of intrinsic activity in these areas during pubertal development. The spatial distribution of these associations is shown in Figure 1.

**Figure 2.**
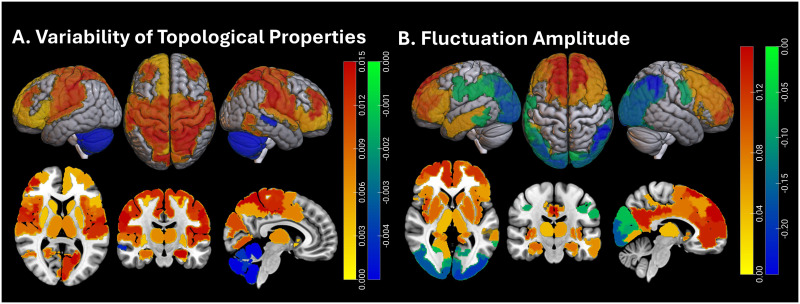
Spatial distribution of significant differences in temporal topological (left panel) and signal variability (fluctuation amplitude; right panel) between girls and boys. The left panel shows regions in which temporal fluctuations of one or more topological parameters were statistically associated with biological sex. Colors correspond to regression coefficients in models testing sex differences in these parameters, with adjustments for other demographic parameters and pubertal stage. Positive values (yellow to red colors) indicate greater variability in girls; and negative values (blue to green colors), lower variability.

Temporal fluctuations of regional topology were also associated with other participant characteristics. The spatial distribution of statistical differences in temporal topological and signal variability between boys and girls is shown in Figure 2, with positive associations reflecting higher temporal parameter variability in girls. Overall, girls had higher variability in local topologies in distributed brain regions, including the somatomotor regions, prefrontal cortex, insula, precuneus, superior temporal gyrus, hippocampus, basal ganglia, and thalamus (*p* < 0.05, *β* = 0.003–0.016, 95% CI = [0.0002, 0.02]), and higher fluctuation amplitude in several of the same regions, including the prefrontal cortex, insula, precuneus, basal ganglia, and thalamus, as well as bilateral cingulate cortices (*p* < 0.04, *β* = 0.04–0.20, 95% CI = [0.003, 0.25]). In addition, girls had lower topological fluctuations primarily in the cerebellum (*p* < 0.05, *β* = −0.005 to −0.004, 95% CI = [−0.01, −0.001]) and lower fluctuation amplitude in posterior occipital, parietal, and (left) temporal regions (*p* < 0.04, *β* = −0.28 to −0.04, 95% CI = [−0.34, −0.003]).

Temporal fluctuations of regional topological properties were not consistently associated with race/ethnicity. However, higher topological fluctuations (i.e., lower temporal consistency) of regional importance in the network (centrality) and lower fluctuations of local clustering were estimated in racial minorities (including Hispanic participants) across distributed brain regions (*p* < 0.04, *β* = 0.004–0.02, 95% CI = [0.002, 0.01]), and lower centrality and fluctuation amplitude were estimated in prefrontal (including orbitorfrontal) regions, bilateral basal ganglia, and the cerebellum (*p* < 0.05, *β* = −0.25 to −0.04, 95% CI = [−0.32, −0.01]).

**Figure 3.**
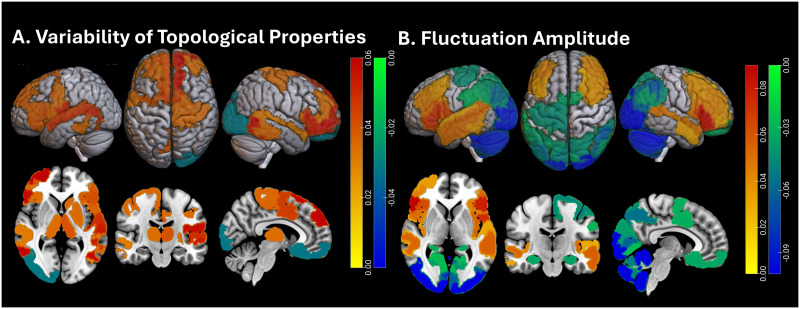
Spatial distribution of regions in which a sex-adjusted BMI *z*-score is statistically associated with fluctuations in topological properties (top) and fluctuation amplitude (bottom). Colors represent standardized regression coefficients, with yellow to red corresponding to positive association and blue to green to negative ones.

In prior analyses of static topological characteristics of resting-state networks in ABCD study samples, BMI has been negatively associated with these characteristics across distributed brain regions and networks (Brooks et al., 2023). In the present dynamic analyses, higher BMI was associated with lower topological fluctuations in not only primary visual areas but also orbitofrontal regions (*p* < 0.05, *β* = −0.04 to −0.03, 95% CI = [−0.07, −0.004]), both parts of the brain where topological fluctuations were not significantly associated with pubertal stage. Higher BMI was also associated with higher topological fluctuations and, thus, lower temporal consistency of topologies in distributed regions, including bilateral temporal regions, bilateral ventral, lateral and superior prefrontal cortices, secondary somatomotor cortex, left precentral gyrus, bilateral insula, right superior temporal gyrus and bilateral basal ganglia, and thalamus (*p* < 0.03, *β* = 0.03–0.06, 95% CI = [0.004, 0.09]). Finally, BMI was also associated with higher fluctuation amplitude in bilateral dorsolateral and ventrolateral prefrontal cortices, insula, and bilateral temporal sulci (*p* < 0.05, *β* = 0.02–0.08, 95% CI = [0.003, 0.10]). Some of these overlapped with areas of higher topological fluctuation as a function of BMI. Finally, BMI was negatively associated with fluctuation amplitude in the bilateral parietal lobule (inferior and superior)—and bilateral posterior visual areas, bilateral precuneus, left somatomotor cortex, right orbitofrontal cortex, bilateral hippocampus, and bilateral cerebellum (*p* < 0.03, *β* = −0.12 to −0.03, 95% CI = [−0.14, −0.003]). Some of these areas, including the orbitofrontal cortex and the cerebellum, overlapped with regions where fluctuation amplitude decreased as a function of pubertal stage. The spatial distribution of positive and negative BMI associations with regional topological and signal variability is shown in Figure 3.

#### Spatial scales of individual networks and the entire connectome.

At the scales of individual networks and the connectome, no topological properties were statistically associated with pubertal stage. However, later pubertal stages were associated with lower fluctuation amplitude in bilateral limbic, salience, and social networks, and similarly in the bilateral thalamus, amygdala, hippocampus, cerebellum, and right basal ganglia. Model statistics are provided in Table 2. In addition, girls had lower fluctuations (higher temporal consistency) of robustness, stability, and fragility in the bilateral reward, right social (and also lower efficiency and global clustering fluctuations in this network), right frontoparietal control, and right prefrontal networks compared with boys (*p* < 0.05, *β* = −0.01 to −0.001, 95% CI = [−0.02, −0.001]). Girls also had higher fluctuation amplitude in bilateral salience, frontoparietal control, default mode (DM), reward, social, and prefrontal networks, as well as subcortical structures including the thalamus, hippocampus, and basal ganglia (*p* < 0.03, *β* = 0.04–0.10, 95% CI = [0.003, 0.15]), but lower fluctuation amplitude in bilateral central and peripheral visual and dorsal attention networks (*p* < 0.04, *β* = −0.20 to −0.04, 95% CI = [−0.25, −0.001]). Furthermore, racial/ethnic minorities had higher topological fluctuations (and thus lower temporal consistency) across multiple networks, including prefrontal, social, DM, and reward networks (*p* < 0.05, *β* = 0.001–0.023, 95% CI = [0.0002, 0.03]), and lower fluctuation amplitude in some of the same networks, including frontoparietal control, DM, reward, social, and prefrontal networks (*p* < 0.03, *β* = −0.12 to −0.03, 95% CI = [−0.16, −0.004]). Finally, BMI was associated with lower topological and higher amplitude fluctuations in salience and social networks (*p* < 0.05, *β* = −0.06 to −0.03, 95% CI = [−0.08, −0.01], and *p* < 0.02, *β* = 0.03–0.06, 95% CI = [0.004, 0.08], respectively).

**Table 2.** Statistics of mixed effects models (based on integrated baseline and follow-up sample, with random intercepts and slopes for participant and ABCD study site, respectively) that assessed relationships between pubertal stage and fluctuations of network topological properties or signal amplitude

**Networks**	**Statistic**	**Summary values across properties**
**Temporal variability of network-level topological properties**		
None		
**Network-level amplitude fluctuation**		
**Left salience**	Standardized *β*	−0.0389
	95% CI	[−0.0742, −0.0037]
	*p* Value	0.0304
**Right salience**	Standardized *β*	−0.0409
	95% CI	[−0.0775, −0.0042]
	*p* Value	0.0288
**Left limbic**	Standardized *β*	−0.0488
	95% CI	[−0.0891, −0.0085]
	*p* Value	0.0177
**Right limbic**	Standardized *β*	−0.0608
	95% CI	[−0.1014, −0.0202]
	*p* Value	0.0033
**Left social**	Standardized *β*	−0.0364
	95% CI	[−0.0723, −0.0001]
	*p* Value	0.0470
**Right social**	Standardized *β*	−0.0592
	95% CI	[−0.0933, −0.0251]
	*p* Value	<0.0001
**Thalamus**	Standardized *β*	−0.0340
	95% CI	[−0.0567, −0.0112]
	*p* Value	0.0034
**Amygdala**	Standardized *β*	−0.0449
	95% CI	[−0.0741, −0.0158]
	*p* Value	0.0025
**Left hippocampus**	Standardized *β*	−0.0622
	95% CI	[−0.0887, −0.0357]
	*p* Value	<0.001
**Right hippocampus**	Standardized *β*	−0.052
	95% CI	[−0.0829, −0.0215]
	*p* Value	*p* < 0.001
**Right basal ganglia**	Standardized *β*	−0.0340
	95% CI	[−0.0639, −0.0042]
	*p* Value	0.0253
**Cerebellum**	Standardized *β*	[−0.0895, −0.0710]
	95% CI	[−0.1279, −0.0302]
	*p* Value	<0.0001

At the scale of the entire connectome, few associations were identified. Girls had lower modularity fluctuations compared with boys (*p* < 0.01, *β* = −0.005, 95% CI = [−0.009, −0.002]), racial/ethnic minorities had lower global efficiency and clustering fluctuations (*p* < 0.02, *β* = −0.02 to −0.01, 95% CI = [−0.025, −0.003]), and BMI was negatively associated with robustness, efficiency, modularity, and stability fluctuations (*p* < 0.03, *β* = −0.03 to −0.05, 95% CI = [−0.08 to −0.006]). Model statistics are provided in Supporting Information Table S2.

### Topological and Amplitude Fluctuations in Individual Assessments

Additional analyses were conducted at individual assessments. Across spatial scales, associations of topological and signal fluctuations within the more limited range of pubertal stages in individual assessments were not consistent. Within the baseline cohort, the pubertal stage was associated with lower temporal fluctuations of local clustering in the right superior parietal lobule (*p* < 0.04, *β* = −0.06, 95% CI = [−0.10, −0.02]) and lower fluctuation amplitude in the left superior temporal gyrus and right cerebellum (*p* < 0.03, *β* = −0.05, 95% CI = [−0.09, −0.01]). Within the 2-year follow-up cohort, the pubertal stage was associated with lower temporal fluctuations of local clustering in the bilateral orbitofrontal cortex (*p* < 0.02, *β* = −0.09 to −0.07, 95% CI = [−0.14, −0.13]); higher fluctuation amplitude in left dorsolateral prefrontal cortex (PFC), bilateral somatomotor cortex, and left posterior cingulate (*p* < 0.02, *β* = [0.06, 0.09], 95% CI = [0.02, 0.14]); and lower fluctuation amplitude in distributed cortical and subcortical regions (*p* < 0.04, *β* = [−0.14, −0.04], 95% CI = [−0.19, −0.001]). Model results are summarized in Table 3.

**Table 3.** Statistics of mixed effects regression models (with a random intercept and slope for ABCD study site) used to investigate relationships between pubertal stage and fluctuations of regional topological properties and amplitude at individual assessments

**Baseline**		**Two-year follow-up**	
**Statistic**	**Value**	**Statistic**	**Value**
**Local clustering**			
Negative correlations (−*β*)	Right superior parietal lobule	Negative correlations (−*β*)	Bilateral orbitofrontal cortex
Standardized β*	−0.0577	Standardized *β*	[−0.0897, −0.0735]
95% CI	[−0.1001, −0.0153]	95% CI	[−0.1397, −0.0208]
*p* Value	0.0382	*p* Value	<0.019
**Regional fluctuation amplitude**			
Negative correlations (−*β*)	Left superior temporal gyrus Right cerebellum	Negative Correlations (−*β*)	Bilateral orbitofrontal cortex
			Bilateral temporal pole
			Bilateral insula
			Bilateral retrosplenial cortex
			Bilateral parahippocampal area
			Bilateral posterior cingulate
			Left precuneus
			Right posterior medial PFC
			Left frontal opercular area
			Bilateral hippocampus
			Bilateral amygdala
			Bilateral thalamus
Standardized β*	[−0.0515, −0.0496]	Standardized *β*	[−0.1366, −0.0382]
95% CI	[−0.0901, −0.0092]	95% CI	[−0.1911, −0.8218]
*p* Value	<0.0324	*p* Value	<0.0435
		Positive correlations (+*β*)	Bilateral dorsolateral prefrontal cortex Bilateral somatomotor cortex
		Standardized *β*	[0.0603, 0.0901]
		95% CI	[0.0158, 0.1401]
		*p* Value	0.0249

* When multiple regions are involved, the range of regression coefficients *β* is provided.

### Associations Between Topological and Amplitude Fluctuations

**Figure 4.**
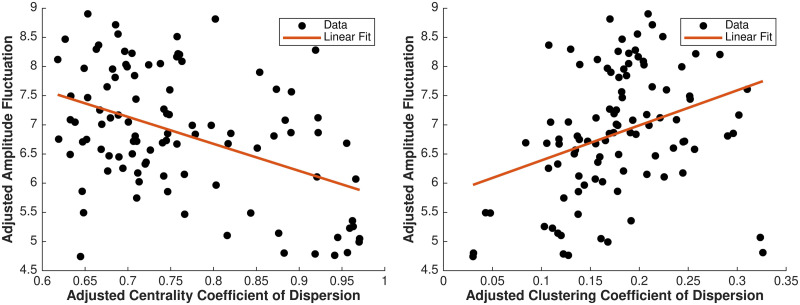
Adjusted region-specific amplitude fluctuation versus adjusted centrality coefficient of dispersion (left panel) and similarly for clustering coefficient of dispersion (right panel). For each of 100 brain regions, the median (over the sample)-adjusted topological property (*x*-axis) is shown as a function of median-adjusted fluctuation amplitude (*y*-axis).

**Figure 5.**
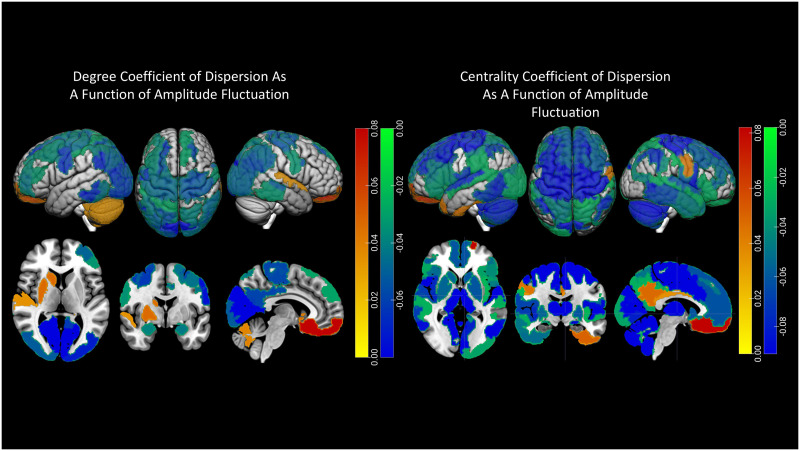
Associations between temporal fluctuations in regional connectedness and fluctuation amplitude (left panel), and temporal fluctuations in regional centrality and fluctuation amplitude (right panel), respectively. Values correspond to standardized regression coefficients for topological fluctuations in models testing their association with fluctuation amplitude. Negative associations are indicated by blue to green colors, and positive associations indicated by yellow to red colors.

Associations between adjusted (for pubertal stage and other individual characteristics) median (over participants) coefficient of dispersion for centrality and amplitude fluctuations, and similarly for clustering coefficient, in the 100 analyzed regions are shown in Figure 4. Higher fluctuations in signal amplitude were associated with lower fluctuations in regional importance in the network (centrality), and similarly for fluctuations in degree, but higher fluctuations in local clustering. Thus, higher fluctuation amplitude was associated with more consistent influence of a region on the connectome, but less consistent connectivity patterns within the region’s neighborhood. The detailed spatial distribution of these associations (positive and negative) is shown in Figure 5 (the left panel shows degree fluctuation [coefficient of dispersion] vs. fluctuation amplitude, the right panel shows centrality fluctuation vs. fluctuation amplitude). These results were replicated based on dynamic topological properties estimated using a longer sliding window and are shown in Supporting Information Figure S1. The identified associations were robust to the choice of window length. For the majority of regions (where associations were statistically significant), the degree coefficient of dispersion was negatively associated with fluctuation amplitude (in distributed posterior, somatomotor, prefrontal, and inferior temporal regions, as well as the bilateral amygdala; *p* < 0.04, *β* = [−0.20, −0.03], 95% CI = [−0.23, −0.002]). In the bilateral orbitofrontal cortex, right superior temporal gyrus, right basal ganglia, left hippocampus, and left cerebellum, the two measures were positively associated (*p* < 0.03, *β* = 0.03–0.09, 95% CI = [0.005, 0.11]). Similarly, the centrality fluctuations were negatively associated with fluctuation amplitude in several distributed brain areas (*p* < 0.05, *β* = [−0.16, −0.02], 95% CI = [−0.18, −0.001]), but positively associated with it in the left orbitofrontal cortex, left temporal pole, left precuneus and posterior cingulate, and right somatomotor area (*p* < 0.02, *β* = [0.04, 0.09], 95% CI = [0.007, 0.12]). These results indicate that across the brain (with few exceptions, especially the orbitofrontal cortex), higher temporal consistency (lower variability) of its topological characteristics is associated with higher regional signal variability.

### Replication Studies

**Figure 6.**
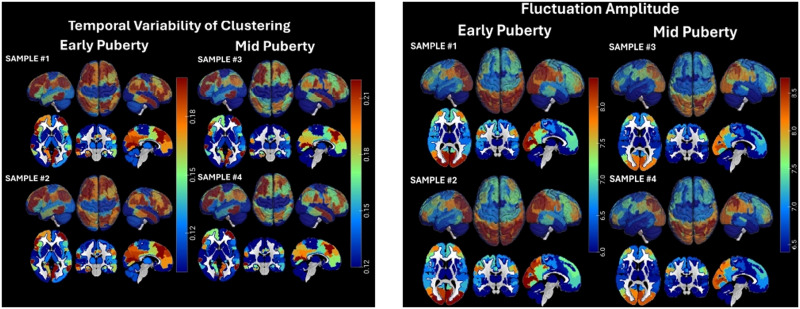
Replication of spatial distributions of topological fluctuations (left panel) and fluctuation amplitude (right panel) in two independent samples in early puberty (Samples #1 and #2), and mid-puberty (Samples #3 and #4). At each region, the median across the sample topological or amplitude variability is shown. All values are positive, with the lowest shown in blue to the highest shown in red.

**Figure 7.**
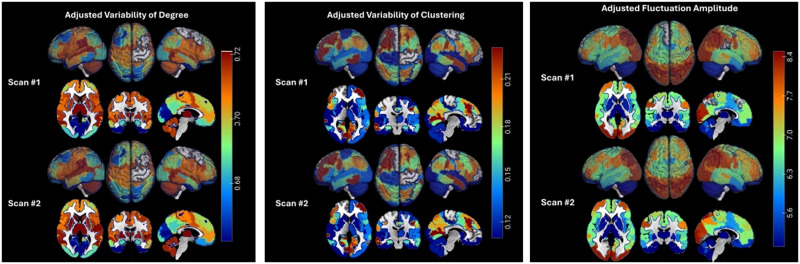
Spatial distribution of topological variability (regional degree and local clustering) and fluctuation amplitude in a subsample of *n* = 2,116 youth with two fMRI scans. Since these scans were on average 2 years apart, variability estimates were adjusted for age, pubertal stage, time of day of scan, and percent of frames censored for motion at the two scans. Values correspond to medians (across each sample) in each region. All values are positive, with the lowest shown in blue to the highest shown in red.

First, dynamic connectivity matrices and topological measures estimated using the original ~9-s sliding window were re-estimated with a 16-s window (20 frames). Supporting Information Figure S2 shows examples of dynamic connectivity matrices (high-resolution and downsampled) estimated at multiple windows, and Supporting Information Table S1 provides statistics on similarity between connectivity matrices of 100 randomly selected participants estimated using three windows (10, 15, and 20 frames). Similarity was high (cosine similarity in range 0.77–0.95) across window comparisons. Statistical analyses of dynamic topologies (based on the longer 16-s window) of the entire samples were then repeated, and relationships between topological and signal fluctuations and pubertal stage were compared between windows. To assess the consistency of topological variability and fluctuation amplitude, these parameters were compared in independent cohorts within the pubertal stage, focusing on early and mid-puberty, since independent samples were larger and, thus, captured higher inherent heterogeneity of the brain and its dynamics. The spatial distributions of these parameters were statistically similar (*p* > 0.10) between samples at each pubertal stage, and are shown in Figure 6. These findings indicate high reproducibility and group-level consistency of estimated parameter dynamics. Finally, in youth with two scans (at baseline and follow-up), estimated topological and signal variability was first statistically adjusted (to account for age and pubertal stage differences, as well as differences in time of scan and number of frames censored for motion) and was then compared between scans. The distributions of estimated degree and clustering fluctuations and fluctuation amplitude across scans were statistically similar (*p* > 0.10) and are shown in Figure 7. Together, these comparisons indicate that dynamic topological and amplitude variability at rest is highly reproducible across samples and scans.

## DISCUSSION

In a sample of over 4,000 adolescents from the ABCD study (including a subsample of over 2,000 youth with early longitudinal data), spanning from pre/early puberty to late/post puberty, this study investigated the pubertal changes of dynamic spontaneous regional coordination patterns and signal fluctuations in the developing brain. It examined fluctuations of resting-state network topological properties and corresponding fluctuation amplitude. Given the dFC thresholding approach in the study, fluctuations of strongest inter-regional connections and their networks were examined (typically top ~10%–20% connections in each frame). Prior work suggests that higher temporal variability of resting-state activity but lower variability of dFC may predict better task performance. Thus, there is a critical link between spontaneous brain dynamics and cognitive function. Studies have correlated both the temporal variability (and complexity) of BOLD signals, and coupling between this variability and topological characteristics of spontaneously coordinated brain networks, with composite cognitive scores, fluid intelligence, and processing speed (Cohen, 2018; Di et al., 2023; Liégeois et al., 2019; Omidvarnia et al., 2021; Sheng et al., 2021; Ye et al., 2024). In children, dynamic topological changes have been associated with specific cognitive states and processes (Marusak et al., 2017). Furthermore, across the adult lifespan, aberrant variability of task-related BOLD activity has been associated with poorer cognitive performance in domain-specific tasks (Boylan et al., 2021). Pediatric studies have also linked specific dFC patterns and associated states with mental health (Fu et al., 2018, 2021, 2025). Despite this body of prior work, only few studies in children have examined age-related changes in dFC (Faghiri et al., 2018; Lei et al., 2022; López-Vicente et al., 2021), and none has specifically focused on puberty, a period of not only profound endocrine changes but also heightened neural maturation and reorganization of brain circuits that support complex cognitive function and mental health. This study has sought to address this significant gap in knowledge.

In spatially distributed brain areas, spontaneous regional connectedness (degree) and importance in the network (which depends on connectedness) were inversely associated with the amplitude of signal fluctuations. Thus, a higher dynamic consistency of a region’s spontaneous coordination with the rest of the brain was associated with higher fluctuations of regional activity. There were some exceptions, particularly in the orbitofrontal cortex, where the opposite associations were observed. These findings suggest that associations between topological and low-frequency signal fluctuations may have a scale and regional dependence. Thus, higher local signal fluctuations in specific but spatially distributed brain regions may be associated with higher temporal consistency of regional topological characteristics relating to not only the large-scale connectome but also the higher variability of local (within community) coordination.

In several brain areas and networks, intrinsic signal variability (measured by fluctuation amplitude) changed as a function of pubertal stage. It increased in not only somatomotor but also lateral prefrontal cortical areas, but decreased in insula, cingulate cortex, orbitofrontal cortex, amygdala, basal ganglia, and hippocampus.

Given the conservative thresholding of dFC, fluctuations of the strongest instrinsic connections were investigated and overlap (at least partly) with the default-mode network, which is active at rest. This network undergoes significant reorganization during development (Fair et al., 2008), but the lack of statistical changes of the network’s intrinsic topology and regional activity as a function of puberty suggests that not only the sparsest but also strongest intrinsic connections could be in place prior to the onset of puberty and/or may be dynamically consistent. High reproducibility of these findings suggest that, indeed, these patterns may be invariant to pubertal stage, samples, and snapshots of brain dynamics captured in short periods of time during a resting-state fMRI scan. Lower fluctuations in local network coordination were associated with higher spontaneous fluctuations in brain activity, indicating a direct mapping between low-frequency activity and local dFC. However, the amplitude of these fluctuations decreased during puberty, suggesting that increased dynamic consistency of intrinsic activity may be a marker of neural maturation.

These findings were robust across spatial scales. Later pubertal stages were associated with lower fluctuation amplitude in bilateral limbic (including that of the cingulate cortex and the amygdala, also found to decrease with pubertal stage), salience and social networks, thalamus, and cerebellum. Overall, these relationships are in agreement with previous findings in adults (Fu et al., 2017), including relatively lower dynamic changes in the cerebellum (Zalesky et al., 2014). Also, identified regions of increasing fluctuation amplitude during puberty overlapped with developmental cortical gradients of intrinsic activity previously reported in youth (from 8 to 18 years; Sydnor et al., 2023). There were also some differences between our findings and those in adults, namely, lower amplitude fluctuations in orbitofrontal regions in youth as a function of pubertal stage and (on average) lower amplitude fluctuations in bilateral limbic networks. The prefrontal cortex undergoes accelerated maturation in adolescence, a process that is also highly heterogeneous. Similarly, limbic networks undergo substantial rewiring during puberty to support emotional processing and regulation (Arain et al., 2013; Sturman & Moghaddam, 2011). It is, therefore, likely that resting-state amplitude fluctuations have distinct trends within puberty, compared with adulthood, especially in underdeveloped brain areas. Furthermore, spatially distributed changes in fluctuation amplitude during puberty may also reflect the extent of maturation in the adolescent brain. Prior studies in adults have identified spatially localized regions of differentially high fluctuation amplitude (Fransson, 2005). This study has only examined youth in puberty, during which neural maturation is spatially distributed, as brain circuits become progressively more specialized locally (to facilitate domain-specific computations in segregated communities), and redundant connections are eliminated while selected connections are strengthened to facilitate integration of domain-specific information (Fair et al., 2007).

Prior work has shown increased coupling between the structural and functional connectome as a function of development (Baum et al., 2020), as well as differential coupling between the two in distinct brain areas in adults (Liu et al., 2022). In adolescence, the prefrontal cortex, limbic, and social networks and their constituent areas undergo accelerated maturation. Progressive optimization of their anatomical connections to improve the efficiency of information processing in the developing brain likely reduces the temporal variability of their intrinsic activity and coordination patterns. However, already developed and likely strongest anatomical connections between regions are less likely to change substantially during puberty, and thus, their intrinsic connection patterns may already be temporally consistent.

Multiple individual factors were associated with pubertal trajectories of dynamic resting-state network and signal fluctuations. Overall, higher signal and topological temporal variability was estimated in girls across spatially distributed regions (with the exception of the cerebellum). These included the prefrontal regions, insula, precuneus, superior temporal gyrus, cingulate cortex basal ganglia, and thalamus, that is, regions at different stages of neural maturation in adolescence. At the network level, lower variability of some of these networks’ topological characteristics and more consistent topological robustness and stability were estimated in girls. Multiple studies have reporter developmental differences in white matter and maturation of brain circuits between girls and boys (Koolschijn & Crone, 2013; Lenroot & Giedd, 2010; Lim et al., 2015) and sex-related differences in the topological organization of distinct circuits (Ingalhalikar et al., 2014). Our results are not only aligned with these findings but also indicate that there may be inherent sex differences in brain dynamics that are independent of pubertal stage.

Some statistical associations between race-ethnicity and signal and topological fluctuations were identified, but were less consistent. In multiple brain networks and regions, associations between topological and signal fluctuations in racial minorities (as a group) were in the opposite direction than those in the entire cohort (which is predominantly White and non-Hispanic), or specifically in White non-Hispanic youth, with higher topological variability (and, thus, lower temporal consistency). Specifically, higher topological fluctuations in prefrontal, social, DM, and reward networks and lower fluctuation amplitude in several of the same networks were estimated. Prior work, including recent studies based on the ABCD cohort, have identified structural and functional differences in brain maturation in racial minorities and their interactions with other environmental and socioeconomic factors (Dumornay et al., 2023; Harnett et al., 2024). These studies have identified specific morphological differences (especially in cortical thickness) between White youth and racial minorities, in developing brain areas, including prefrontal cortical regions involved in emotion regulation. These regions overlap with those identified in the present study, which suggests potential associations between morphological and dynamic alterations in racial minorities.

Finally, higher BMI was associated with higher topological fluctuations and, thus, lower temporal consistency of topologies and signal amplitude in distributed regions, including developing prefrontal regions, the insula, and the superior temporal gyrus, but also parts of the somatomotor cortex, the basal ganglia, and the thalamus. Large-scale studies, including our prior work on the baseline ABCD cohort (i.e., a subset of this study’s sample), have identified widespread negative associations between BMI and fundamental morphometric and topological characteristics of developing brain networks in early adolescence, overlapping with those identified in the present study (Brooks et al., 2023; Dennis et al., 2022; Laurent et al., 2020). These findings suggest that BMI-related differences in intrinsic brain dynamics may be associated with underlying morphological differences.

Despite its many strengths, this study also had some limitations. Although standard for 3.0 T scanners, the resting-state fMRI (rs-fMRI) sampling rate was relatively low (TR = 0.8 s), which implies dynamics at shorter temporal scales could not be resolved. This is a common limitation of most fMRI studies examining BOLD dynamics with widely used scanners. There are ongoing efforts to obtain progressively higher temporal resolution (and robust) rs-fMRI (e.g., TR = 0.5 s in 7 T scanners; Yoo et al., 2018), but these scanners are not being used by the ABCD. Another limitation is that estimates of temporal signal and/or network fluctuations in subcortical structures that are harder to image, especially in children, may be less reliable than those in cortical structures. However, the best-quality fMRI run based on multiple criteria was selected for analysis, all data were harmonized across scanners to eliminate scanner-related differences, all time-series were carefully preprocessed, and frames were censored for motion and artifacts using conservative thresholds and were excluded from analysis if they did not meet these thresholds. Also, dynamic connectivity matrices were estimated using multiple distinct methodologies to assess similarity of topological patterns as a function of the selected method. Furthermore, this study used a very conservative dFC threshold, which preserved the strongest, and likely most developed, intrinsic connections. A future study could examine topological dynamics using less-conservative thresholds to resolve additional networks with dynamic topological properties and signal variability that may change substantially with age. In addition, as the longitudinal data of the ABCD study grow, additional assessments will facilitate investigations of developmental changes in intrinsic dynamics over longer periods of time. Finally, we examined puberty trajectories of temporal variability measures as a function of demographic parameters and BMI. Although a wide range of environmental, behavioral, and other individual factors may impact these trajectories, an exhaustive investigation of their impact on these trajectories was beyond the scope of the study and could be the focus of future work.

Despite a few limitations, this study makes a significant scientific contribution toward our incomplete understanding of developmental changes in the dynamics of spontaneous activity and coordination in the adolescent brain. It has identified primarily inversely related fluctuations in topological properties and low-frequency signals across the brain and has shown that neural maturation during puberty may be associated with increased consistency of spontaneous activity in select but spatially distributed brain regions, including those that undergo significant reorganization during adolescent development. These regions overlap with brain networks that support cognitive functions that change significantly in adolescence, including emotional processing and regulation (supported by limbic and salience networks and the amygdala), reward processing, and social function (supported by the social brain—a set of distributed regions and networks with overlapping roles). Prior work has characterized these fluctuations as a fundamental property of multiscale brain organization (Baracchini et al., 2023) and has correlated them with cognitive performance across domains. Findings from our study also suggest that maturation (including myelination) of the underlying anatomical connections, and thus the structural constraints of functional networks, may be driving these changes (Wang et al., 2021). The study has also identified sex differences in these changes, which are aligned with prior work showing faster maturation of brain circuits in girls during puberty, and also racial/ethnic differences, suggesting potential maturation disparities in racial/ethnic minorities. Finally, findings also suggest that excess BMI may have detrimental effects on the trajectories of resting-state signal and network dynamics. As the ABCD study continues to collect longitudinal data, these two markers of brain development can be tracked over time in the same participants to assess their trajectories into late adolescence and potential modulation by individual and environmental factors.

## MATERIALS AND METHODS

### Participants

The analytic sample included *n* = 4,099 youth from the baseline assessment (53.3% female, median age = 120.0 months, IQR = 13.0 months) and *n* = 3,376 youth from the 2-year follow-up (51.1% female, median age = 144 months, IQR of 13 months). A subsample (*n* = 2,116) had early longitudinal fMRI data, that is, at both baseline and follow-up. Details on participant/data exclusion to minimize confounding effects of factors that may impact intrinsic network activity are provided in Brooks et al. (2021). Briefly, youth with diagnosed neurodevelopmental disorders, schizophrenia, bipolar disorder, and/or clinical findings in their structural MRI were excluded to minimize the potential confounding effects of these disorders and/or structural anomalies on topological and dynamic brain measures of interest. In addition, information provided by the ABCD study on quality control of the neuroimaging data and exclusion criteria set by our laboratory on data contamination by movement in the scanner were used to further exclude participants.

### fMRI Data Processing

The study analyzed rs-fMRI from the ABCD longitudinal study cohort, Release 4.0. These data had been minimally preprocessed by the Data Analysis, Informatics & Resource Center of the ABCD study (Hagler et al., 2019) and were further processed using tools from the custom Next-Generation Neural Data Analysis (NGNDA) pipeline. All neuroimaging data had been collected across 21 sites, in 3.0 T GE, Siemens, or Philips scanners. The TR for fMRI (2.4 mm isotropic) was 0.8 s. Processing using the NGNDA included coregistration to structural MRI, normalization to MNI152 space, motion correction, frame removal and interpolation to exclude artifact-contaminated frames, signal denoising, and signal amplitude harmonization across scanners (Brooks et al., 2021). Voxel-level signals were downsampled to 1,088 parcels using high-resolution cortical (1,000 parcels), subcortical, and cerebellar atlases (Diedrichsen et al., 2009; Schaefer et al., 2018; Tian et al., 2020). The resting-state fMRI protocol of the ABCD study includes up to four 5-min long runs (almost all participants had 5-min runs, with <0.5% having shorter or longer runs). The NGNDA pipeline excludes runs with more than 10% of frames censored for motion (assuming a displacement cutoff of 0.3 mm). Thus, participants who do not have at least one run with less than 10% censored frames are excluded from further analysis. In this study, for each participant, their best-quality fMRI was selected for analysis. This run typically had the lowest number of frames censored for motion (median = 1.9% at baseline and 1.3% at follow-up) and lowest median resting-state connectivity, estimated from the time-compressed data, assuming that the brain at rest is overall weakly correlated. The latter criterion was imposed to further reduce the likelihood of including runs with spuriously high correlations between brain regions. At baseline, participants were scanned from 8 am to 8 pm, with median time of scan (IQR in hr) = 14:00 (4). At follow-up, they were scanned from 8 am to 9 pm, median = 13:00 (4).

### Dynamic Functional Network Estimation

Dynamic fluctuations of the connectome’s topological properties and signal amplitude were estimated from parcellated rs-fMRI time-series, with each parcel corresponding to a network node. Analyses were conducted at three spatial scales: individual regions (nodes), large resting-state networks, and the entire connectome (brain-wide).

**Figure 8.**
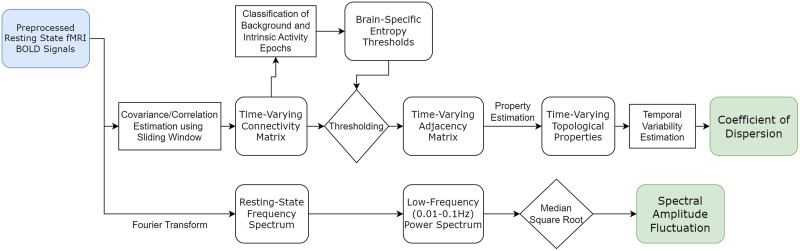
Flow chart of methodology used to estimate the topological property and amplitude fluctuations.

The analytic approach used in the estimation of dynamic topologies and signal fluctuations is summarized in Figure 8. Using an 8.8-s (11 frames) sliding window with a one time point (frame) increment, dynamic covariance matrices were estimated and transformed to correlation matrices (size 1,088 × 1,088 at each time point). The window size was selected after correlation matrices estimated with several windows (5, 15, and 20 frames long; see examples in Supporting Information Figure S2) were compared, based on assumptions of statistical stationarity. For replication purposes, a longer sliding window (20 frames, 16.0 s) was also used, and dynamic topological properties and their variability were re-estimated and analyzed as a function of pubertal stage for replication purposes. A number of other approaches have been previously used to estimate dynamic connectivity: Some are based on an assumption of locality, using statistical modeling in windows with longer time scales (40 s or more) and tapered shapes, while other approaches are window independent (Allen et al., 2014; Fu et al., 2021; Yaesoubi et al., 2018). To assess method dependence, a second method was used to estimate these matrices, based on an instantaneous phase. Each fMRI time-series was transformed to a complex-valued signal via the Hilbert transform, from which an instantaneous phase of each time point was estimated. The absolute difference in the instantaneous phase between pairs of signals, scaled to a range of [−1, 1], was used as another measure of time-dependent connectivity. Prior work has reported statistical similarities between connectivity matrices estimated using this approach and window-based methods. In this sample, moderate topological similarity between connectivity matrices calculated by the two methods was estimated (median Baroni-Urbani [Baroni-Urbani & Buser, 1976] index = 0.67). Results in this study are based on the covariance-based approach. Dynamic topological property estimation in large samples is computationally expensive. To facilitate tractable analyses at multiple spatial scales, higher resolution dynamic connectivity matrices (1,088 × 1,088 per frame) were downsampled to a lower resolution (100 × 100 per frame) based on anatomical considerations and spatial locations of 89 cortical regions and 11 subcortical (amygdala, thalamus, basal ganglia, and hippocampus) and cerebellar regions. Examples of high-resolution and downsampled connectivity matrices are shown in Supporting Information Figure S3. The 17 cortical networks identified in Yeo et al. (2011), and additional reward network (including the medial prefrontal cortex, ventral striatum, orbitofrontal cortex, anterior cingulate, and amygdala [Haber & Knutson, 2010]), social network (which supports social function and includes the medial prefrontal cortex, temporoparietal junction, inferior frontal gyrus, interparietal sulcus, anterior cingulate cortex, anterior insula, and the amygdala [Blakemore, 2008]), and prefrontal networks were then analyzed. The prefrontal cortex, reward network, and social network undergo heightened maturation in adolescence, but their dynamic topological properties have not been systematically investigated. Thus, it was important to analyze their dynamics as a function of pubertal stage. These networks are composed of distributed regions, some of which overlap across these three networks and the 17 cortical networks.

#### Entropy-based thresholding.

Multiple strategies have been previously proposed for thresholding connectivity matrices, including dynamic ones, based on cohort or brain-specific statistics, such as absolute or proportional thresholds (Garrison et al., 2015; van den Heuvel et al., 2017; Xia et al., 2019), data-driven thresholds derived using minimal spanning trees (Dimitriadis et al., 2017), or other approaches, such as incorporating spatial fluidity to accommodate network variation in space and time (Iraji, Deramus, et al., 2019).

At rest, the brain is typically weakly correlated, with the exception of epochs of spontaneous coordination. These shifts in functional connectivity may be associated with shifts in entropy (which can be used to quantify signal/image complexity). In the prior work, regional signal complexity at low frequencies (<0.1 Hz) has been associated with higher functional connectivity (Liu et al., 2019; Wang et al., 2018). In this study, entropy of regional correlations was estimated in order to derive brain-specific thresholds for dynamic connectivity matrices. Specifically, at each time point, the approximate entropy of each region’s correlation with all others was estimated, resulting in 100 entropy values. Their dimension across time and space (100 × 375 values for a 5-min rs-fMRI run) was then reduced using a principal component analysis. Sensitivity analyses were conducted to assess differences in resulting thresholds as a function of the number of selected principal components. Differences in the thresholds depending on the number of principal components used was very small (median difference was 0.2% of the threshold [IQR = 0.5%]). Thus, 10 components were selected (which explained on average ~60% of the variability of time-dependent entropy values). Then, *k*-means clustering of the lower dimension data was used to identify clusters of entropy values across time. A minimum *k* = 3 was set, assuming that there are at least three distinct dynamic connectivity clusters: one corresponding to a state of weakly correlated brain regions at rest (background), one associated with spontaneous brain coordination, and a high correlation cluster corresponding to spuriously correlated regions, likely due to movement in the scanner or other artifacts. The elbow method was then used to determine the optimum number of clusters. In both baseline and follow-up samples, median (IQR) optimal *k* was 3 (2) (maximum = 12 clusters). Examples of the number of clusters as a function of the within-cluster sum of squared distances of data points from the cluster centroid are shown in Supporting Information Figure S4. Given the overall weak inter-regional correlations at rest, it was assumed that the cluster with the highest membership included entropy values related to this state. Corresponding correlation values in that cluster were then used to estimate a brain-specific threshold. A conservative brain-specific threshold was calculated as the moderate outlier of the absolute correlation values in the background cluster. If this value was >0.9 (suggesting implausibly high background connectivity), the 75th percentile of the absolute correlation values was instead used as the threshold.

#### Estimation of temporal variability of topological properties.

Time-varying topological properties were calculated from adjacency matrices at each time point. These properties includes median connectivity, community structure (modularity), global, network-wide and local clustering, topological efficiency (Rubinov & Sporns, 2010), network fragility (Pasqualetti et al., 2020), network robustness and resilience (Wu et al., 2011), and regional importance in the network (centrality) and connectedness (degree). Together, these properties describe the instantaneous organization of the connectome.

For each brain and time-dependent topological property, values corresponding to frames censored for motion and time points where the density of the adjacency matrix density was higher than the extreme outlier of all densities across time were excluded from further calculations, as they were likely contaminated by artifacts and/or spurious correlations introduced by excessive motion in the scanner. The temporal fluctuation of each property across the remaining time points was calculated as the quartile coefficient of dispersion—the IQR divided by the sum of the first and third quartiles, given the non-normal distribution of time-dependent topologies.

### Estimation of Resting-State Signal Fluctuation Amplitude

Developmental changes in the temporal variability of low-frequency (≤0.1 Hz) rs-fMRI signal amplitude have recently been reported (Syndor et al., 2023). Following a similar approach, the spectrum of each fMRI parcel signal was calculated, and the median square root of the frequency content in the range 0.01–0.10 Hz was estimated as the fluctuation in signal amplitude. Parcel-level (node) vectors were downsampled to 100 region-level vectors, by taking the median of fluctuation amplitude over parcels within each region. In addition, the median over nodes in each of the analyzed networks was also calculated to assess network-level dynamic amplitude fluctuations.

### Statistical Analysis

Regression models were developed to investigate topological property and amplitude fluctuations at the regional, network, and whole-brain spatial scales, as a function of development, that is, pubertal stage (provided on a scale 1–5, with 1 = prepuberty and 5 = postpuberty). The ABCD uses the Pubertal Development Scale (Peterson, 1988), for this purpose. Pubertal stage is calculated based on parent responses to questions on their child’s height spurt, skin changes, body hair, breast development, menstruation, deepening voice, and facial hair, which are summed and then categorized. Since <1% of youth were in postpuberty, for modeling purposes they were combined with those in late puberty in a single category. In addition to separate models used to examine developmental changes within assessments, a set of mixed effects models were also developed, to include data from both assessments and a random intercept and slope for participant, to account for their repeated data (baseline and follow-up). For *n* = 45 participants (0.84% of the sample), pubertal stage at follow-up was earlier than baseline, which is biologically implausible. These participants were excluded from final analyses.

All analyses were adjusted for sampling differences across sites, using propensity weights provided by the ABCD. All developed models were linear mixed effects regression models that included the following covariates: age at scan, sex assigned at birth, race-ethnicity, family income, BMI *z*-score (stratified by sex), scan hour (prior work has shown correlations between resting-state topological characteristics and time of scan (Hu et al., 2023)), and percent of fMRI frames (relative to scan length) censored for motion and ABCD site (models included a random intercept and slope for site). The significance level was set at 0.05, and all *p* values were corrected for the false discovery rate (FDR), using established approaches (Benjamini & Hochberg, 1995). At the whole-brain and network scales, FDR corrections were done across topological properties. At the scale of individual regions, FDR corrections were done across regions within an individual network. When appropriate, regression coefficients were standardized. All analyses were performed in the Harvard Medical School High Performance Cluster, using MATLAB (release R2023a).

### Replication Analyses

Three types of replication analyses were conducted. In the first analysis, a longer sliding window (12 s long [20 frames]) was used to re-estimate dynamic connectivity matrices and their topological properties at the three spatial scales of interest, in the entire baseline and follow-up samples. Statistical analyses examining associations with pubertal stage were then replicated. Also, in a smaller cohort of *n* = 100 participants, the topologies of high-resolution and downsampled dynamic connectivity matrices estimated using sliding windows of three different sizes (10, 15, and 20) were compared. Examples and similarity statistics are provided in Supporting Information (Figure S2 and Table S1). The second analysis identified two independent subsamples in early puberty (*n* = 826 and 626, respectively) and similarly for mid-puberty (*n* = 838 and 931, respectively), and compared the spatial distribution of topological fluctuations and signal fluctuation amplitude in these samples within each pubertal stage, to assess their similarity. The third analysis was based on the sample of *n* = 2,116 with longitudinal data. Age and pubertal stage effects were regressed out, and the distributions of topological fluctuation and signal fluctuation amplitude of the same set of participants with two scans were then compared.

## ACKNOWLEDGMENTS

This study was conducted with support from the National Science Foundation, Awards #1940096, #2116707, and #2207733.

## SUPPORTING INFORMATION

Supporting information for this article is available at https://doi.org/10.1162/netn_a_00452.

## AUTHOR CONTRIBUTIONS

Jethro Lim: Formal analysis; Investigation; Methodology; Software; Visualization; Writing – original draft; Writing – review & editing. Kaitlynn Cooper: Formal analysis; Methodology; Software; Writing – review & editing. Catherine Stamoulis: Conceptualization; Funding acquisition; Investigation; Methodology; Project administration; Resources; Supervision; Writing – original draft; Writing – review & editing.

## FUNDING INFORMATION

Catherine Stamoulis, National Science Foundation (https://dx.doi.org/10.13039/100000001), Award ID: 1940096. Catherine Stamoulis, National Science Foundation (https://dx.doi.org/10.13039/100000001), Award ID: 2116707. Catherine Stamoulis, National Science Foundation (https://dx.doi.org/10.13039/100000001), Award ID: 2207733.

## DATA AND COMPUTER CODES AVAILABILITY

This study analyzed publicly available data from the ABCD data, which are available at the National Institute for Mental Health data repository: https://nda.nih.gov/.

All computer codes are available in a dedicated GitHub: https://github.com/cstamoulis1/Topological_Signal_Dynamics_in_Puberty/.

## Supplementary Material



## TECHNICAL TERMS

Fluctuation amplitude: Median square root of the BOLD signals’ frequency content in the 0.01- to 0.1-Hz range.
Mixed effects regression model: Model that contains both fixed and random effects (a random intercept and slope) to account for stratification according to specific grouping and/or repeated data.
