# Supplementary material for: Dynamic fluctuations of intrinsic brain activity are associated with consistent topological patterns in puberty and are biomarkers of neural maturation
Source: Netw Neurosci. 2025 Sep 19;9(3):1039–64. doi: 10.1162/netn_a_00452 (PMC12548668; doi:10.1162/netn_a_00452)
Supplement: Supplementary file 1 [file netn-9-3-1039-s001.pdf]

SUPPLEMENTAL MATERIALS

TABLES

Table S1: Cosine similarity of dynamic connectivity and adjacency matrices calculated using a 10-frame long window versus longer windows, across 100 randomly selected participants.

**Comparison**	**Median (IQR) of Subject Median-Over-Time Cosine Similarity**
**High-Resolution Dynamic Connectivity**	
**Length comparison: 10 vs 15**	0.8754 (0.0190)
**Length comparison: 10 vs 20**	0.7677 (0.0339)
**Length comparison: 15 vs 20**	0.9208 (0.0164)
**Downsampled Dynamic Connectivity**	
**Length comparison: 10 vs 15**	0.9159 (0.0278)
**Length comparison: 10 vs 20**	0.8424 (0.0474)
**Length comparison: 15 vs 20**	0.9498 (0.0206)

Table S2: Statistical models on associations between fluctuations in topological properties and sex (modeled as male = 1, female = 2), race-ethnicity (modeled as white non-Hispanic = 0, others = 1), and BMI (z-score stratified for sex). All reported p-values have been adjusted for the False Discovery Rate (FDR). When appropriate, regressions coefficients have been standardized.

STATISTIC	SUMMARY VALUES ACROSS PROPERTIES
**CONNECTOME-WIDE**	
**SEX ASSIGNED AT BIRTH**	
Topological Properties	Modularity
Beta	-0.0055
95% CI	-0.0090 to -0.0021
P-Value	0.012
**RACE-ETHNICITY (WHITE NON-HISPANIC vs RACE/ETHNIC MINORITIES)**	
Topological properties	Efficiency, clustering
Beta	-0.0155 to -0.0104
95% CI	-0.0254 to -0.0032
P-Value	<0.016
**BMI (Z-SCORE)**	
Topological properties	Efficiency, modularity, robustness, stability
Beta	-0.0528 to -0.0323
95% CI	-0.0794 to -0.0059
P-Value	< 0.029

**NETWORK-SPECIFIC**	
**SEX ASSIGNED AT BIRTH**	
**Topological Robustness**	
Negative Correlations (-$\boldsymbol{\beta}$)	Bilateral Reward<br>Right Social<br>Right Frontoparietal Control<br>Right Prefrontal Cortex
Beta	-0.0107 to -0.0084
95% CI	-0.0162 to -0.0026
P-Value	<0.020
**Topological Stability**	
Negative Correlations (-$\boldsymbol{\beta}$)	Bilateral Reward<br>Right Social<br>Right Prefrontal Cortex
Beta	-0.0063 to -0.0051
95% CI	-0.0098 to -0.0015
P-Value	< 0.020
**Topological Fragility**	
Negative Correlations (-$\boldsymbol{\beta}$)	Bilateral Reward<br>Right Social<br>Right Frontoparietal Control
Beta	-0.0025 to -0.0015
95% CI	-0.0040 to -0.0002
P-Value	< 0.043
**RACE-ETHNICITY<br>(WHITE NON-HISPANIC vs RACIOETHNIC MINORITIES)**	

**Within-Network Median Connectivity**	
Positive Correlations (+β)	Right Default Mode<br>Bilateral Dorsal Attention<br>Bilateral Prefrontal Cortex<br>Left Salience<br>Right Reward<br>Bilateral Social<br>Bilateral Somatomotor
Beta	0.0011 to 0.0022
95% CI	0.0003 to 0.0034
P-Value	<0.047
**Between-Network Median Connectivity**	
Positive Correlations (+β)	Bilateral Somatomotor
Beta	0.0010 to 0.0011
95% CI	0.0002 to 0.0019
P-Value	< 0.030
**Network Robustness**	
Positive Correlations (+β)	Bilateral Somatomotor
Beta	0.0081 to 0.0108
95% CI	0.0030 to 0.0159
P-Value	< 0.01
**Network Efficiency**	
Positive Correlations (+β)	Bilateral Somatomotor
Beta	0.0122 to 0.0232

95% CI	0.0023 to 0.0343
P-Value	< 0.030
Negative Correlations (-β)	Left Reward
Beta	-0.0086
95% CI	-0.0147 to -0.0025
P-Value	0.045
**Modularity**	
Negative Correlations (-β)	Right Reward
Beta	-0.0104
95% CI	-0.0172 to -0.0035
P-Value	0.024
**Network Fragility**	
Positive Correlations (+β)	Right Somatomotor
Beta	0.0034
95% CI	0.0005 to 0.0063
P-Value	<0.028
**BMI (z-score)**	
**Topological Robustness**	
Negative Correlations (-β)	Bilateral Social<br>Right Reward
Beta	-0.0551 to -0.0350

95% CI	-0.0815 to -0.0085
P-Value	<0.026
**Network Global Clustering**	
Negative Correlations (-$\boldsymbol{\beta}$)	Right Reward<br>Right Social
Beta	-0.0429 to -0.0331
95% CI	-0.0695 to -0.0067
P-Value	< 0.028
**Network Stability**	
Negative Correlations (-$\boldsymbol{\beta}$)	Left Salience Ventral Attention<br>Right Reward<br>Right Social
Beta	-0.0575 to -0.0363
95% CI	-0.0839 to -0.0094
P-Value	<0.032
**Network Fragility**	
Negative Correlations (-$\boldsymbol{\beta}$)	Left Dorsal Attention<br>Bilateral Reward<br>Bilateral Social<br>Bilateral Salience Ventral Attention
Beta	-0.0510 to -0.0369
95% CI	-0.0777 to -0.0105
P-Value	< 0.0490

Table S3: Statistics of models testing associations between network-level fluctuation amplitude and sex (modeled as male = 1, female = 2), race-ethnicity (modeled as white non-Hispanic = 0, others = 1), and BMI (z-score stratified for sex). All reported p-values have been adjusted for the False Discovery Rate (FDR). When appropriate, regressions coefficients have been standardized.

**STATISTIC**	**SUMMARY VALUES**
**NETWORK-SPECIFIC**	
**SEX ASSIGNED AT BIRTH**	
Positive correlations (+β)	Bilateral Salience<br>Bilateral Frontoparietal Control<br>Bilateral Default Mode<br>Bilateral Reward<br>Bilateral Social<br>Bilateral Prefrontal Cortex<br>Bilateral Thalamus<br>Bilateral Hippocampus<br>Bilateral Basal Ganglia
Beta	0.0360 to 0.1042
95% CI	0.0035 to 0.1480
P-Value	< 0.03
Negative correlations (-β)	Bilateral Visual (Central)<br>Bilateral Visual (Peripheral)<br>Bilateral Dorsal Attention
Beta	-0.1981 to -0.0386
95% CI	-0.2493 to -0.0015
P-Value	< 0.04
**RACE-ETHNICITY (WHITE NON-HISPANIC vs RACIOETHNIC MINORITIES)**	
Positive correlations (+β)	Left Visual (Peripheral)<br>Bilateral Somatomotor
Beta	0.0589 to 0.0700

95% CI	0.0061 to 0.1169
P-Value	< 0.030
Negative correlations (-$\boldsymbol{\beta}$)	Bilateral Dorsal Attention<br>Left Limbic<br>Bilateral Frontoparietal Control<br>Bilateral Default Mode<br>Bilateral Reward<br>Bilateral Social<br>Right Salience Ventral Attention<br>Bilateral Prefrontal Cortex<br>Bilateral Basal Ganglia<br>Bilateral Cerebellum
Beta	-0.1191 to -0.0349
95% CI	-0.1591 to -0.0042
P-Value	< 0.032
**BMI (Z-SCORE)**	
Positive correlations (+$\boldsymbol{\beta}$)	Left Frontoparietal Control<br>Bilateral Salience Ventral Attention<br>Bilateral Temporo-parietal<br>Bilateral Reward<br>Bilateral Social<br>Bilateral Default Mode<br>Bilateral Prefrontal Cortex
Beta	0.0279 to 0.0600
95% CI	0.0039 to 0.0845
P-Value	<0.024
Negative correlations (-$\boldsymbol{\beta}$)	Bilateral Visual (Central)<br>Bilateral Visual (Peripheral)<br>Bilateral Dorsal Attention<br>Bilateral Hippocampus<br>Bilateral Cerebellum
Beta	-0.1158 to -0.0318
95% CI	-0.1398 to -0.0096

P-Value	<0.01

FIGURES

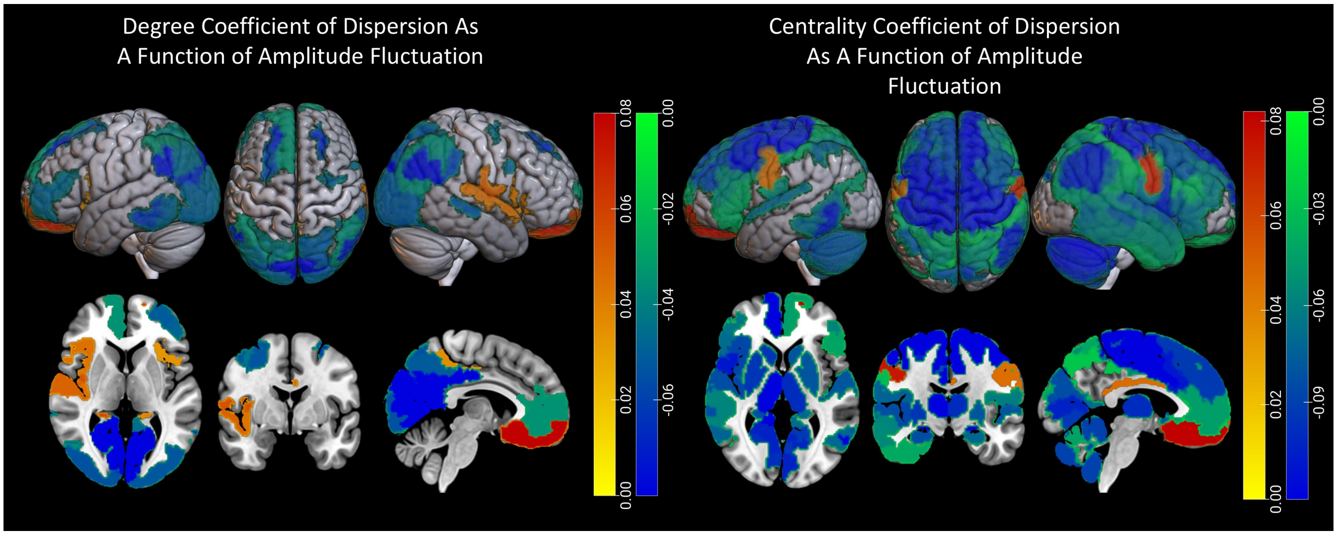

Figure S1: Associations between temporal fluctuations in regional connectedness and fluctuation amplitude (left panel), and temporal fluctuations in local clustering and fluctuation amplitude (right panel), respectively. Values correspond to standardized regression coefficients for topological fluctuations in models testing their association with fluctuation amplitude. Negative associations are indicated by blue to green colors, and positive associations by yellow to red colors. Dynamic topological properties were estimated using a 16.0 s sliding window.

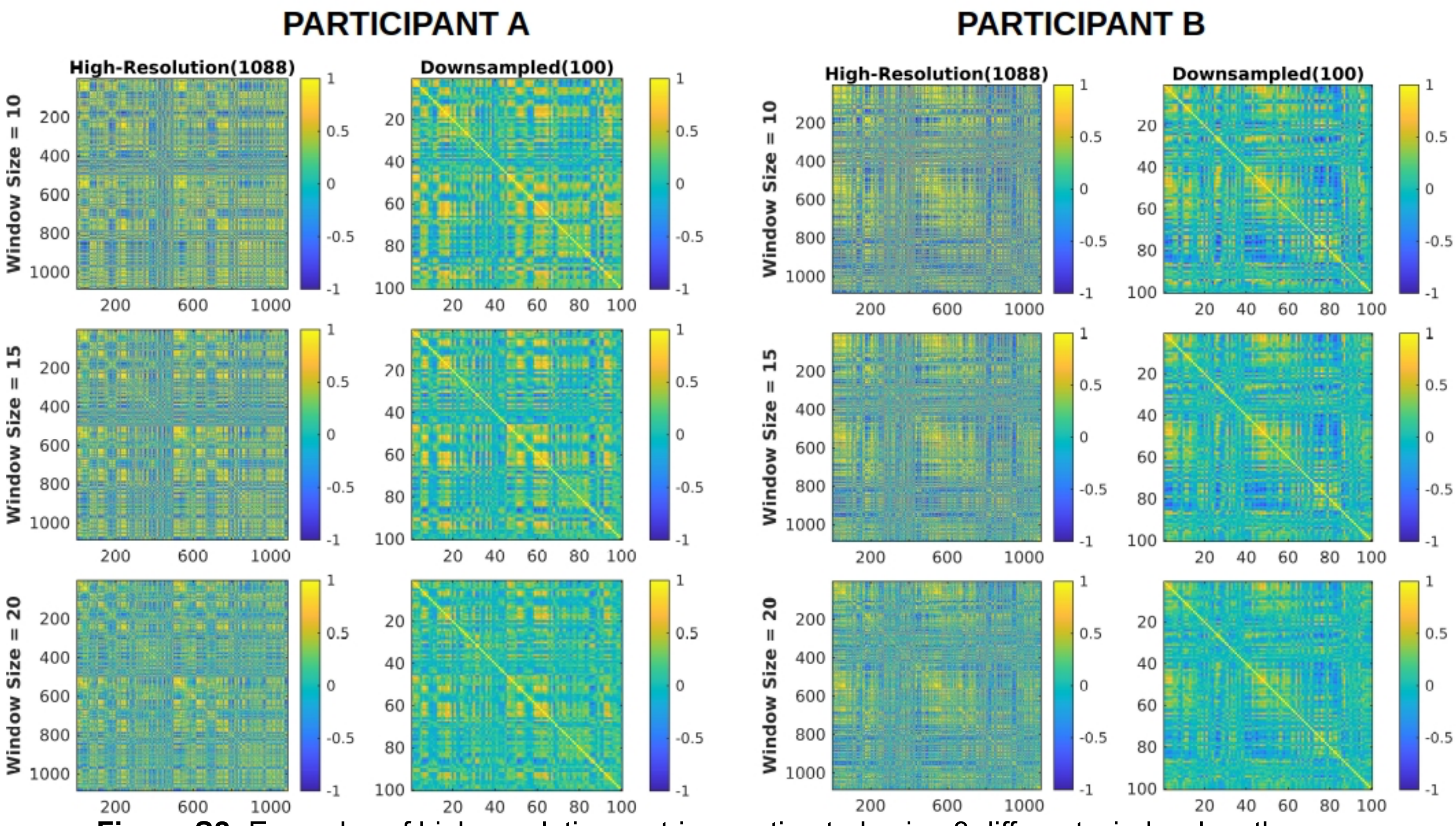

Figure S2: Examples of high-resolution matrices estimated using 3 different window lengths (10, 15 and 20 frames) and their downsampled counterparts from two participants (A and B).

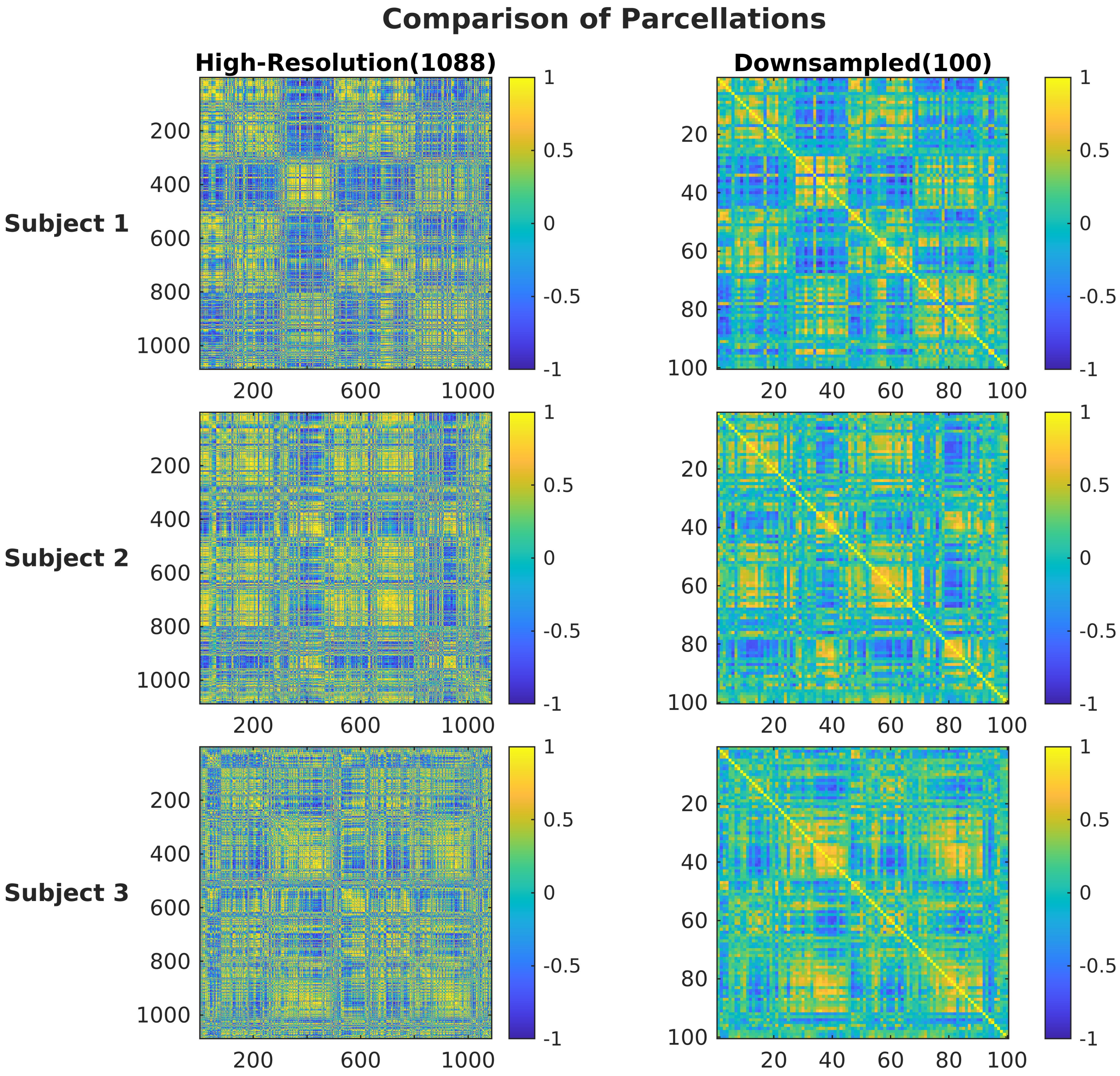

Figure S3: Examples of instantaneous high-resolution (left column) and downsampled (right column) connectivity matrices estimated using a 10 frame sliding window, from three participants.

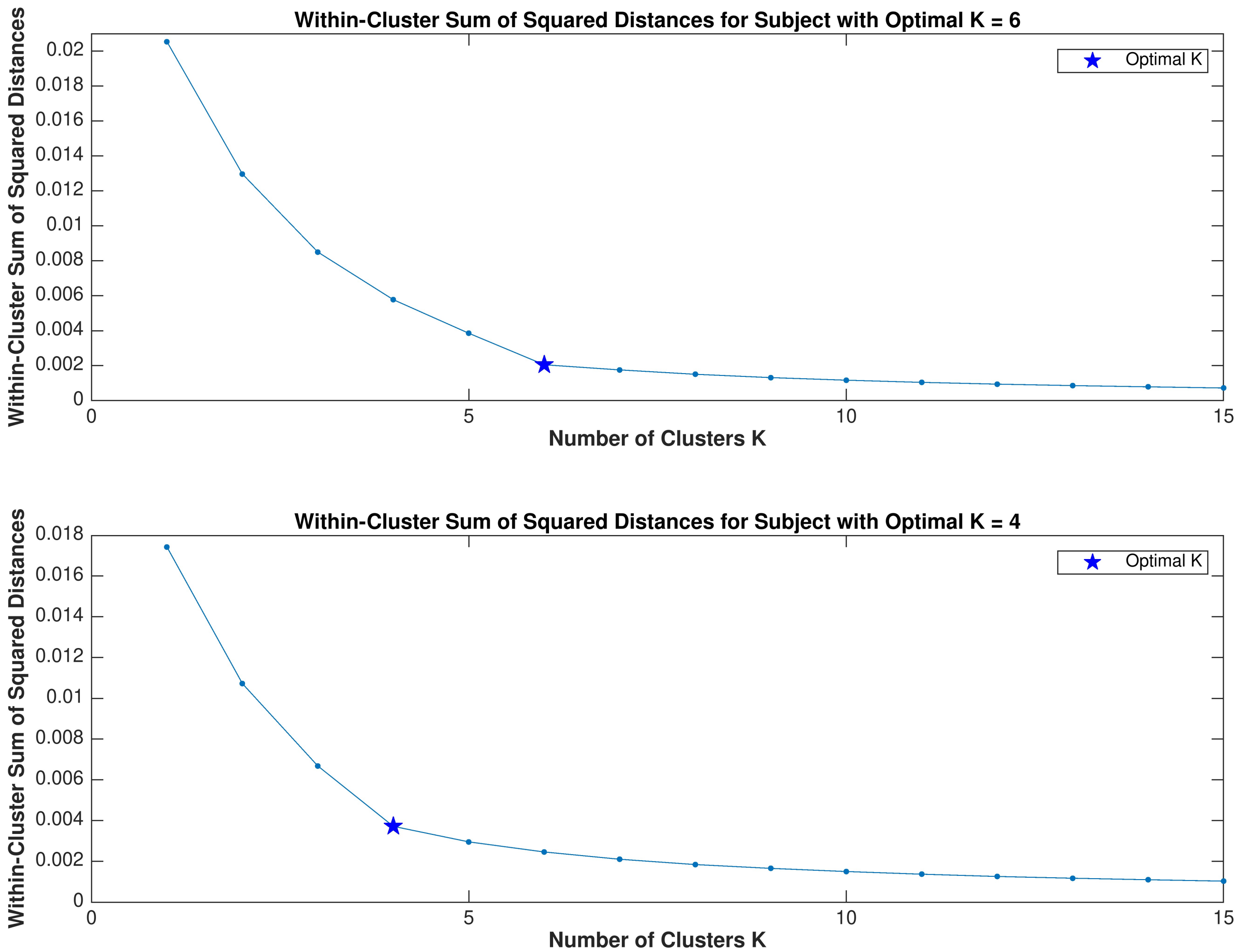

Figure S4: Examples of the elbow method used to choose the number of connectivity clusters used in the estimation of threshold used to obtain time-varying adjacency matrices from connectivity matrices. The examples are from two participants and show within cluster sum of squared distances (from the cluster centroid) as a function of number of clusters obtained via the k-means classification method. The elbow is indicated by the star, and corresponds to the optimal number of clusters.
